# Opinion: Methodological Shortcomings in the Study on a Prophage-based PCR Test for Lyme Borreliosis

**DOI:** 10.3389/fmicb.2021.802131

**Published:** 2021-12-13

**Authors:** Freek R. van de Schoor, M. E. Baarsma, Mariska M. G. Leeflang, Volker Fingerle, Gabriele Margos, Joppe W. Hovius, Alje P. van Dam

**Affiliations:** ^1^Radboudumc, Department of Internal Medicine, Radboudumc Center for Infectious Diseases and Radboud Institute of Health Sciences, Nijmegen, Netherlands; ^2^Center for Experimental and Molecular Medicine, Amsterdam Institute for Infection and Immunology, Amsterdam UMC, University of Amsterdam, Amsterdam, Netherlands; ^3^Epidemiology and Data Science, Amsterdam Public Health, Amsterdam UMC, University of Amsterdam, Amsterdam, Netherlands; ^4^German National Reference Centre for Borrelia, Bavarian Health and Food Safety Authority, Oberschleißheim, Germany; ^5^ESCMID Study Group for Lyme Borreliosis, Basel, Switzerland

**Keywords:** *Borrelia burgdorferi*, Lyme borreliosis, PCR, diagnosis, prophage

We read the article by Shan et al. (2021) with great interest, as new diagnostic tests for Lyme borreliosis (LB) are urgently needed (Cruickshank et al., 2018; Dessau et al., 2018). The article represents a proof of principle paper and an initial validation of an already commercially available test [Phelix Phage Borrelia—R.E.D. Laboratories (redlabs.be)]. We have various concerns regarding the study design, novelty of the approach, technical aspects of the assay, statistical analyses, and the conclusions, which must be addressed. Of note, several statements in the introduction are speculative and not supported by the references, but unfortunately, the word limit of our opinion does not allow us to elaborate on this.

## Gene sequences and PCR results

**Figure 1:**
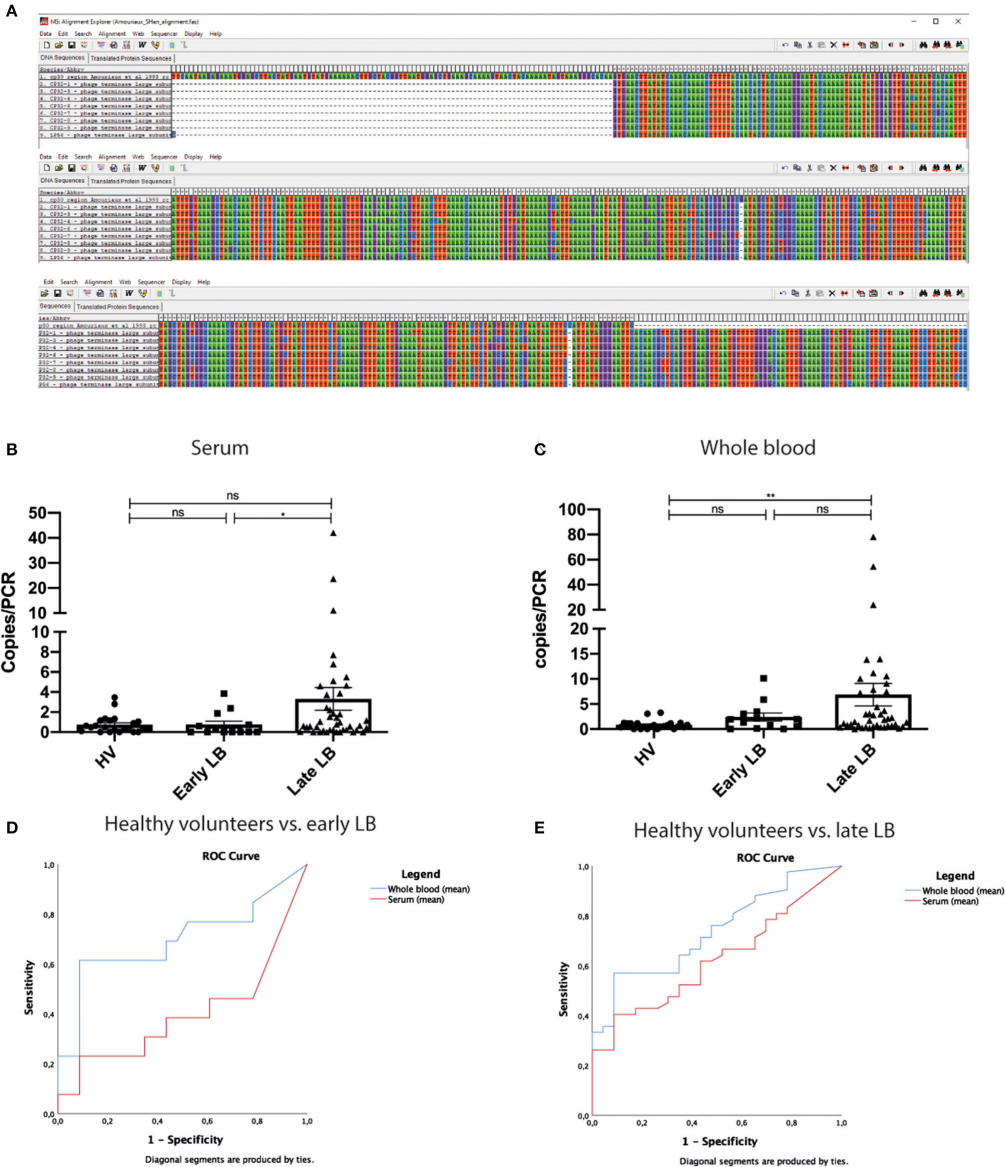
Alignment, individuals values of copies/PCR, and ROC analysis of copies/PCR. **(A)** Sequences published by Amouriaux et al. (1993) and by Shan et al. (2021) (termed cp32-1 to cp32-9 and lp56, lines 2–9) were aligned in MEGA 5 (Tamura et al., 2011). The alignment shows that there is some overlap between these sequences. The overlap starts at position 102 into the sequence published by Amouriaux et al. (1993) (first line) and continues until pos 420. Dashes indicate the end of sequence used by Amouriaux. There are only two mismatches to cp32-1 and these are single base insertions. These data indicate that the cp32 terminal phage subunit gene region was already used as a target for diagnostic *Borrelia* PCR prior to the publication by Shan et al. **(B,C)** Even though the mean value of copies/PCR are significantly different in our analysis between late LB and healthy individuals, there is significant overlap between all groups. This was observed both in serum and in WB. **(D,E)** ROC curves of mean values WB and serum. **(C)** Comparison of healthy volunteers to early LD. Whole blood AUC = 0.697(95%CI 0.496–0.899). Serum AUC = 0.400 (95%CI 0.187–0.612). **(D)** Comparison of healthy volunteers and late LD. Whole blood AUC = 0.738 (95%CI 0.618–0.858). Serum AUC = 0.622 (95%CI 0.487–0.757). LB, Lyme borreliosis; WB, whole blood; ROC, receiver operating characteristic; AUC, area under the curve; CI, confidence interval; ns, non-significant; *p < 0.05, **p < 0.01, calculated using independent-samples Mann-Whitney U-test for comparing participant groups; dependent-samples Wilcoxon Signed Rank test for comparing whole-blood vs. serum within a participant group.

The concept of targeting genetic material from bacteriophages rather than from bacteria for clinical diagnosis is intriguing and—while not entirely new—it is still relevant today (Amouriaux et al., 1993). In a previous publication, Amouriaux et al. (1993) describe a similar approach targeting a plasmid region with sequence overlap to sequences used in the current publication (Figure 1A). However, the authors do not prove that bacteriophages are present and circulating in human blood. Therefore, the difference in sensitivity between the 16S PCR and *terL* PCR could actually be due to the difference in sensitivity between using a single-copy (16S) and a multi-copy (*terL*) target. This principle is well-known in bacteriology (Roosendaal et al., 1993). In addition, the genetic variation between and within the *Borreliella burgdorferi* sensu lato (s.l.) species of cp32 bacteriophage sequences is not discernible from the manuscript. In the alignment shown in Figure 2 in the article by Shan et al., the authors use cp32 genes of *B. burgdorferi* sensu stricto (s.s.)., but not of other species. *B. burgdorferi* s.s. B31 has the highest number of cp32 (*n* = 13) in comparison to 16S rRNA, a single copy chromosomal locus. We would be very interested to see how *Borrelia afzelii* and *Borrelia garinii* would have performed in analyses using spiked blood samples, as these are the most common genospecies causing clinical symptoms in Europe, but have—according to the authors' Table 1—fewer cp32 plasmids (*n* = 8, *n* = 4, respectively). In their paper, the geographical origin of patients is not described, but the authors state that “*patients were diagnosed by Dr. LT*,” referring to Louis Teulières, who is based in Paris, France, where—like in the rest of the European continent—LB is caused mainly by *B. afzelii* and *B. garinii* (Stanek et al., 2012). However, the test method is based on the *terL* gene derived from the North American strain of *B. burgdorferi* s.s. B31. Furthermore, the authors include extremely low-positive signals in their results. Of the 23 healthy controls, 21 showed a positive signal in the *terL* PCR in at least one of the 12 samples. Whereas incidental carriage of *Borrelia-*DNA in blood of healthy persons, as suggested by the authors, might occur, it is highly unlikely that this would be found in over 90% of the population. This strongly suggests that at least some low-positive results represent unspecific signals or signals which are a result of DNA cross-contamination.

## Selection Criteria LB Patients

Another concern is the patient selection and interpretation of the clinical data. The manuscript lacks any description of patient characteristics, and does not report inclusion or exclusion criteria. Absence of clear eligibility criteria may indicate selection bias. Criteria for patient selection in an LB-related diagnostic test accuracy study should be clear and unambiguous, for example based on European guidelines (Mygland et al., 2010; Stanek et al., 2011; Hofmann et al., 2017). The authors refer to the ILADS guideline (Cameron et al., 2014), which in itself does not contain any diagnostic criteria. Without unambiguous criteria, one cannot ensure that these individuals were in fact patients with LB (Stanek et al., 2011; Lantos et al., 2021). It is also unclear what is meant by “early LD” and “late LD.” Would Lyme neuroborreliosis (LNB) be classified as early or late LD, for example (Koedel and Pfister, 2017)? Were there any LNB patients at all? If so, how were they diagnosed?

## Statistical Analysis

We attempted to replicate the analyses presented by the authors in their Figure 7, using SPSS version 25. The authors describe having used Mann-Whitney U-tests to compare early LB patients, late LB patients, and healthy volunteers (HVs). However, they do not describe precisely how the results from the different groups have been compared. Their original dataset contains six test results on whole blood (WB) and six on serum for each participant, but it is unclear whether they analyzed all results, if they analyzed the mean per participant or used any transformation of the data. We replicated the Mann-Whitney U-tests to test for a difference between the different participant groups, using the mean values of the six iterations of each test per participant. While the authors' reported means and the means calculated by us were identical, our *p*-values were inconsistent with those reported by the authors. Subsequent analyses using other aggregate functions (such as medians) as input for our statistical tests did not result in *p*-values consistent with those reported by the authors either (data not shown). In contrast, when we used the six iterations of the *terL* assay per participant separately, as if they were independent values, the levels of statistical significance match those reported by the authors. By doing so the authors seem to have artificially inflated their statistical power by increasing their sample size six-fold. This may have resulted in identical mean values, but incorrect and much lower *p*-values. More so, the results from serum and WB samples from one participant are not independent, as both measurements were done in the same person. Therefore, a Wilcoxon Signed-Rank test would have been more appropriate to compare serum and WB within one patient group. The authors do not describe what statistical test they used, but if this was a Mann-Whitney U-test as described in the Methods section, then this is inappropriate.

## The Conclusions are Not Supported by the Data

The aforementioned considerations cast substantial doubt on the reliability of the results, but—when interpreted with caution—do not undermine the value of the authors' hypothesis. Unfortunately, the conclusions drawn by the authors from the results are inappropriate. The authors state that their assay can distinguish early LB, late LB, and HVs. These conclusions are not supported by the data.

The mean/median copy numbers may be significantly different at a group level—even though we have shown in this manuscript's Figures 1B,C and Supplementary Table 1 that they are not for most comparisons—but that does not imply diagnostic power. Only if there is little or no overlap between numeric values, will the assay be able to distinguish a patient from a non-patient. A simple scatterplot of the data shows there is a high degree of overlap between the groups. Subsequent ROC-analysis Figures 1C,D on the mean/median copy numbers shows that—when a minimally acceptable specificity of 90% is applied—the maximally attainable sensitivity is 62% (WB-MEAN: HV vs. early, cutoff at 1.275) or 57% (WB-MEAN: HV vs. late, cutoff at 1.283). This is worse than single-tier or modified two-tiered testing (MTTT) serology in EM and far worse than any type of serology in late LB (Leeflang et al., 2016; Waddell et al., 2016; Branda et al., 2017). Additional ROC-analyses show that the ability to discriminate between early vs. late LB is even (much) worse (data not shown). Please note that these analyses were performed with a small number of samples (early: *n* = 13; late: *n* = 42; HV: *n* = 23). It is much more likely that the assay lacks specificity and that many HVs had false-positive results, rather than suffer from asymptomatic *B. burgdorferi* infection, as the authors claim.

We must also point out that the manuscript suffers from flawed circular reasoning and over-interpretation. The fact that the groups differ with respect to the primary study parameter does not prove that they are LB patients or HVs. Participants' status as belonging to either group is the starting point for investigating potential differences in *terL* levels, not a conclusion that can be drawn when these groups are indeed shown to be different on this outcome. The authors postulate that their test could be used to monitor LB treatment outcomes, yet, this study does not report on any follow-up samples or treatment outcome to support this claim. They further state that the Ter-qPCR could be used to indicate which treatment option may work best, however, the choice of treatment option is not supported by any of the data in this article.

## Conclusions

We conclude that while this technique might be promising, the paper provides more questions than answers and contains a large number of inaccuracies. We would be interested to see the Ter-qPCR be validated on a cohort of clearly described LB patients and healthy controls from both North America and Europe before we could draw any conclusions on the diagnostic performance of the Ter-qPCR.

## Author Contributions

FS and MEB: conceptualization, methodology, software, formal analysis, resources, and writing—original draft. FS, MEB, and GM: investigation. FS, MEB, ML, VF, GM, and JH: writing—review and editing. JH: supervision and funding acquisition. All authors contributed to the article and approved the submitted version.

## Funding

FS, MEB, and JH are funded by the Netherlands Organization for Health Research and Development (ZonMw, Project Number 522050001). JH's work was partially funded through the European Regional Development Fund and the Interreg North Sea Region Programme 2014–2020 as part of the NorthTick project (Reference Number J-No: 38-2-7-19).

## Conflict of Interest

MEB and JH: LB diagnostics in collaboration with various companies, although none of that work involved molecular detection of *B. burgdorferi* sensu lato. MEB and JH have not received any personal compensation from any of said companies, nor were any of said companies involved in any aspect of the current manuscript. VF: Research support: RKI/BMG, ESCMID, ECDC, StMGP/StMUG, INSTAND. Lecturing activities (honoraria, travel expenses): DIAMEDIS, Diasorin, Mikrogen, Seramun, Siemens, HLR. Consulting activities EQA schemes (no honoraria): QCMD, INSTAND, ECDC. The remaining authors declare that the research was conducted in the absence of any commercial or financial relationships that could be construed as a potential conflict of interest.

## Publisher's Note

All claims expressed in this article are solely those of the authors and do not necessarily represent those of their affiliated organizations, or those of the publisher, the editors and the reviewers. Any product that may be evaluated in this article, or claim that may be made by its manufacturer, is not guaranteed or endorsed by the publisher.
