# Supplementary material for: Opinion: Methodological Shortcomings in the Study on a Prophage-based PCR Test for Lyme Borreliosis
Source: Front Microbiol. 2021 Dec 13;12:802131. doi: 10.3389/fmicb.2021.802131 (PMC8710760; doi:10.3389/fmicb.2021.802131)
Supplement: Supplementary file 1 [file Table_1.docx]

**Supplemental Table 1**

|  | **Shan et al.** | | | | **Reanalyses for the current manuscript** | | | |
| --- | --- | --- | --- | --- | --- | --- | --- | --- |
| **HV vs. early LB** | | | | | | | | |
|  | *HV: mean* | *Early LB: mean* | ***p-value*** | *HV: mean* | | *Early LB: mean* | ***p-value*** |  |
| Whole blood | 0.8 | 2.4 | <0.05 | 0.82 | | 2.41 | 0.051 |  |
| Serum | 0.6 | 0.8 | N/A | 0.74 | | 0.75 | 0.328 |  |
| **HV vs. late LB** | | | | | | | | |
|  | *HV: mean* | *Late LB: mean* | ***p-value*** | *HV: mean* | | *Late LB: mean* | ***p-value*** |  |
| Whole blood | 0.8 | 6.9 | <0.0001 | 0.82 | | 6.87 | 0.002 |  |
| Serum | 0.6 | 3.3 | N/A | 0.74 | | 3.32 | 0.104 |  |
| **Early LB vs. late LB** | | | | | | | | |
|  | *HV: mean* | *Late LB: mean* | ***p-value*** | *HV: mean* | | *Late LB: mean* | ***p-value*** |  |
| Whole blood | 2.4 | 6.9 | <0.01 | 2.41 | | 6.87 | 0.394 |  |
| Serum | 0.8 | 3.3 | N/A | 0.75 | | 3.32 | 0.039 |  |

**Supplemental Table 1: Re-analyses of differences in *terL* levels between groups**. Mean values of copies numbers were recalculated with the data provided by the authors in the Supplementary Material applying the Mann-Whitney U test. We found various discrepancies in p-values.

LB = Lyme borreliosis; HV = healthy volunteers; N/A = not available.
